# Ancient pathogen-driven adaptation triggers increased susceptibility to non-celiac wheat sensitivity in present-day European populations

**DOI:** 10.1186/s12263-016-0532-4

**Published:** 2016-05-23

**Authors:** Marco Sazzini, Sara De Fanti, Anna Cherubini, Andrea Quagliariello, Giuseppe Profiti, Pier Luigi Martelli, Rita Casadio, Chiara Ricci, Massimo Campieri, Alberto Lanzini, Umberto Volta, Giacomo Caio, Claudio Franceschi, Enzo Spisni, Donata Luiselli

**Affiliations:** 1 Laboratory of Molecular Anthropology, Department of Biological, Geological and Environmental Sciences, 40126 Bologna, Italy; 2 Centre for Genome Biology, Department of Biological, Geological and Environmental Sciences, 40126 Bologna, Italy; 3 Department of Biological, Biocomputing Group, Geological and Environmental Sciences, University of Bologna, 40126 Bologna, Italy; 4 CIRI Health Science and Technologies, University of Bologna, 40064 Ozzano dell’Emilia, Bologna, Italy; 5 Department of Clinical and Experimental Sciences, Gastroenterology Unit, Spedali Civili, University of Brescia, 25123 Brescia, Italy; 6 Department of Medical and Surgical Sciences, Digestive Diseases and Internal Medicine Unit, St. Orsola-Malpighi Hospital, University of Bologna, 40138 Bologna, Italy; 7 Department of Experimental, Diagnostic and Specialty Medicine, University of Bologna, 40126 Bologna, Italy; 8 Department of Biological, Unit of Gut Physiopathology and Nutrition, Geological and Environmental Sciences, University of Bologna, 40126 Bologna, Italy

**Keywords:** Non-celiac wheat sensitivity, Human dietary shifts, Human adaptation, Natural selection, Evolutionary medicine

## Abstract

**Background:**

Non-celiac wheat sensitivity is an emerging wheat-related syndrome showing peak prevalence in Western populations. Recent studies hypothesize that new gliadin alleles introduced in the human diet by replacement of ancient wheat with modern varieties can prompt immune responses mediated by the CXCR3-chemokine axis potentially underlying such pathogenic inflammation. This cultural shift may also explain disease epidemiology, having turned European-specific adaptive alleles previously targeted by natural selection into disadvantageous ones.

**Methods:**

To explore this evolutionary scenario, we performed ultra-deep sequencing of genes pivotal in the CXCR3-inflammatory pathway on individuals diagnosed for non-celiac wheat sensitivity and we applied anthropological evolutionary genetics methods to sequence data from worldwide populations to investigate the genetic legacy of natural selection on these loci.

**Results:**

Our results indicate that balancing selection has maintained two divergent *CXCL10/CXCL11* haplotypes in Europeans, one responsible for boosting inflammatory reactions and another for encoding moderate chemokine expression.

**Conclusions:**

This led to considerably higher occurrence of the former haplotype in Western people than in Africans and East Asians, suggesting that they might be more prone to side effects related to the consumption of modern wheat varieties. Accordingly, this study contributed to shed new light on some of the mechanisms potentially involved in the disease etiology and on the evolutionary bases of its present-day epidemiological patterns. Moreover, overrepresentation of disease homozygotes for the dis-adaptive haplotype plausibly accounts for their even more enhanced CXCR3-axis expression and for their further increase in disease risk, representing a promising finding to be validated by larger follow-up studies.

**Electronic supplementary material:**

The online version of this article (doi:10.1186/s12263-016-0532-4) contains supplementary material, which is available to authorized users.

## Background

Non-celiac gluten sensitivity was defined as a new independent and emerging wheat-related syndrome with respect to celiac disease (CD), irritable bowel syndrome (IBS), or wheat allergy (WA) [1, 2], showing a rapidly evolving trend especially in the Western world [1, 3, 4]. Recently, other wheat proteins in addition to gluten have been proposed to play a role in the development of such pathogenic condition so that it has been suggested to re-classify it as non-celiac wheat sensitivity (NCWS) [5]. Nevertheless, it remains to be elucidated whether the substantial rise of disease prevalence in Western populations [3, 6] is the result of increased awareness and reporting/diagnosis or is actually due to its increasing diffusion in such human groups.

NCWS still has an undefined etiology, and its diagnosis is based on the exclusion of IgE-mediated WA and of CD by negative serological CD markers (i.e., anti-endomysium antibodies, anti-EmA and anti-tissue transglutaminase antibodies, and anti-tTG) [1]. However, although biomarkers of immune response to gluten (e.g., IgG anti-gliadin antibodies, AGA) can be observed in 56–66 % of NCWS cases, a pivotal role of innate immunity in the development of the disease is widely accepted [7, 8].

The role that wheat (*Triticum* spp.) proteins play in determining our health has been accurately dissected, and different studies have shown that CD has increased two- to fourfold over the last 50 years [9, 10]. The causes of this recent CD increase have not been fully determined, but several authors have suggested that the last six decades of industry-driven breeding produced wheat varieties with more reactive proteins [11, 12]. This hypothesis is highly consistent with overexpression of CXCL10 (a CXCR3 ligand) induced in peripheral blood mononucleated cells from NCWS patients by contact with proteins of modern wheat, but not by contact with proteins of ancient wheat varieties [13]. In addition to this, it has been reported that different Th1-associated interferon gamma (IFN-γ) expression is present in NCWS with respect to CD [14]. Interestingly, several IFN-γ-related chemokine ligands bind also to the CXCR3 receptor, playing a key role in the perpetuation of inflammation [15], and the whole CXCR3 axis has been found to be significantly overexpressed in inflammatory bowel disease and other inflammatory phenotypes [16, 17]. Moreover, some single nucleotide polymorphisms (SNPs) at the related genes were found to exert protein expression-regulating effects that can lead to altered IFN-γ pathway [17–19]. Since CXCR3 has been proposed to bind also gliadins [20], it could be hypothesized that gluten itself may trigger an initial innate challenge able to further induce secretion of CXCR3 chemokine ligands and to establish a vicious cycle that results in amplified Th1-type inflammation.

According to this evidence, variation at genes playing a pivotal role in the CXCR3 inflammatory pathway might contribute to disease etiology, albeit no studies have investigated this issue so far, thus preventing identification of possible NCWS genetic determinants. For this purpose, and to contribute to the dissection of NCWS’s main causes and pathogenic mechanisms, we aimed at providing new insights into the evolutionary history of such disease by applying anthropological genetics methods. The rationale underlying this approach moves from the observation that even if NCWS prevalence is still far from being accurately determined, it substantially varies among human groups with different ancestry, with peaks of 3–6 % reported by Italian and US referral centers for gluten-related disorders [3, 21]. In some populations, NCWS thus would occur up to six times more than CD, which shows a prevalence of approximately 1 %. This suggests that various selective pressures having acted on diverse human groups, and in different ways during their early and recent evolutionary history, might explain high and changing worldwide NCWS prevalence. Certainly, such epidemiological pattern is in part due to the different extent of cereal consumption in diverse human societies (i.e., divergent degree of exposure to gluten), although the ever-increasing globalization of human diets should have reshaped this picture towards reduced inter-population differences in disease prevalence. The fact that NCWS occurrence still remains considerably higher in Western populations suggests that its susceptibility extensively varies among human groups also as a consequence of their genetic background. In particular, it can be hypothesized that populations of European ancestry retain NCWS risk variants at appreciable frequency in their gene pools plausibly due to the adaptive role exerted by these alleles in their early history. In fact, by modulating inflammatory reactions, these variants could have favored adaptation of these human groups to highly challenging pathogen landscapes, such as those characterizing the European continent, especially after Neolithic transition [22]. The very recent replacement of ancient wheat with modern dwarf varieties highly selected for improving industrial productivity might have then represented a sudden dietary shift that has turned these past genetic adaptations into disadvantageous maladaptive traits, triggering increased susceptibility of present-day Europeans to NCWS. In fact, different from what occurred in the last 8500 years of human wheat cultivation, contemporary breeding programs have been strongly addressed to enhance gluten strength and viscoelasticity in order to enable dough to tolerate high stresses during mixing and leavening mechanized processes [23, 24]. This gain in wheat technological properties was thus achieved to meet industrial requirements and by specifically selecting protein quality in terms of gliadin and glutenin allelic variants, rather than by considering wheat toxic potential [25]. Unfortunately, the selected protein profiles are characterized by specific immune-stimulatory epitopes that have been proved to cause more substantial immunological responses (e.g., increased chemokine secretion and mucosal immune cells infiltration) with respect to those related to wheat ancestors or early domesticated species [13, 26–29].

To explore this complex scenario and the mechanisms potentially underlying the described maladaptive process, we investigated the genetic legacy of natural selection on major genes involved in the CXCR3 inflammatory pathway in several worldwide populations as well as in a well-characterized sample of NCWS subjects. This enabled to pinpoint past pathogen-driven adaptive events that could have made individuals of European ancestry more prone to the side effects related to the consumption of modern wheat varieties. Therefore, the obtained results promise to contribute to shed light on some of the main mechanisms plausibly responsible for NCWS etiology, as well as on the evolutionary bases of its present-day epidemiological patterns.

## Methods

Schematic representation of the implemented research approach is described as a flowchart detailing the adopted steps for NCWS sample selection as well as the population genetic analytical workflow aimed at identifying signatures of past natural selection that could be implicated in increased disease susceptibility of some present-day human populations (Additional file 1: Figure S1).

### NCWS samples

The present study involved 18 unrelated individuals (i.e., 14 females and 4 males with a median age of 38 years and range of 22–56 years) diagnosed as having NCWS after a thorough evaluation. All subjects were of Italian origins, and their genetic ancestry was further tested by evaluating concordance of ancestry components at the examined genes with those observed in Western European populations. They complained of one or more gastrointestinal (e.g., bloating, abdominal pain, diarrhea/constipation, nausea, epigastric pain, gastro-esophageal reflux, and aphthous stomatitis) and extra-intestinal (e.g., tiredness, headache, joint/muscle pain, arm numbness, “foggy mind,” dermatitis/skin rash, anxiety, depression, and anemia) symptoms with an early onset (i.e., a few hours or days) after gluten ingestion. CD was ruled out in all patients by negativity for tTG and EmA, as well as by the absence of villous atrophy in the duodenal biopsy (Marsh classification 0 or 1), whereas WA was excluded by negativity for specific IgE antibodies to wheat and/or skin prick tests. An open oral wheat challenge was then performed to confirm the clinical suspicion of NCWS.

Informed consent was obtained from all individual participants included in the study, and the study was designed in accordance with the ethical standards of the local independent institutional ethical committee and with the Helsinki Declaration and its later amendments or comparable ethical standards.

### Investigated genomic regions

Genes playing a pivotal role in the CXCR3 chemokine ligand-dependent inflammatory pathway have been searched by literature survey and by exploring known and predicted functional interactions between the CXCR3 receptor and all its possible ligands reported in the STRING database v.9.1 [30], finally focusing on loci characterized by extremely high confidence scores (CS) (i.e., CS > 0.99). Accordingly, the *CXCR3*, *CXCL9*, *CXCL10*, and *CXCL11* genes were selected and their genomic coordinates were submitted to the Ion AmpliSeq™ Designer tool (Life Technologies, Grand Island, NY, USA). This approach enabled to design two different primer pools for the amplification of a genomic interval covering 14.8 kb and including the exonic, intronic, and 2-kb 5′ and 3′ flanking regions of the examined genes.

### DNA libraries’ preparation and sequencing

DNA was extracted from NCWS blood samples using the QIAamp DNA kit (QIAGEN GmbH, Hilden, Germany) and quantified with the Qubit® dsDNA BR Assay Kit (Invitrogen™ Life Technologies, Carlsbad, CA, USA) for Qubit 2.0 Fluorometer.

Library preparation was carried out using the designed primer pools and the Ion AmpliSeq Library Kit 2.0 (Life Technologies, Grand Island, NY, USA), according to the manufacturer’s instructions and using 10 ng of DNA. Ligation of barcodes on the 110 obtained amplicons was performed by means of the Ion Xpress™ Barcode Kit (Life Technologies, Grand Island, NY, USA), whereas Agencourt® AMPure® XP Reagent Kit (Beckman Coulter, Beverly, MA, USA) and Ion Library Quantitation Kit (Life Technologies, Grand Island, NY, USA) were used to purify and quantify amplicons by means of qPCR with a StepOnePlus™ Real-Time PCR System (Life Technologies, Grand Island, NY, USA).

Purified fragments were diluted to 8 pM, pooled, and used to prepare DNA templates with the Ion PGM™ Template OT2 200 Kit (Life Technologies, Grand Island, NY, USA) on an Ion OneTouch™ 2 System (Life Technologies, Grand Island, NY, USA). Template-positive ion sphere particles (ISPs) were then retrieved and submitted to template enrichment using Dynabeads® MyOne™ Streptavidin C1 magnetic beads (Life Technologies, Grand Island, NY, USA) on an Ion OneTouch™ ES instrument (Life Technologies, Grand Island, NY, USA) according to the manufacturer’s instructions.

Enriched DNA libraries were finally sequenced on an Ion PGM™ platform (Life Technologies, Grand Island, NY, USA) by means of two Ion 314™ Chip runs and using the PGM™ 200 Sequencing Kit v.2 (Life Technologies, Grand Island, NY, USA).

### Sequence alignment and variant calling

The TMAP tool of the Torrent Suite™ v. 4.0.2 (Life Technologies, Grand Island, NY, USA) was used to perform polyclonal, low-quality and primer dimer quality control (QC) filters, as well as barcode splitting and primers trimming, and to map the high-quality sequence reads to the reference sequences of each examined gene, thus producing the BAM files used for subsequent analyses.

SNPs and insertion/deletion (INDELs) calling was carried out using the *germ-line* pipeline of the Ion Torrent Variant Caller Plugin v. 4.0 (Life Technologies, Grand Island, NY, USA) that implements a frequentist approach for dealing with high coverage (>60×) genomic positions and a Bayesian method for dealing with loci showing low to medium coverage (10–50×).

In order to control for false-positive calls, especially INDELs, resulting from sequencing errors due to the presence of homopolymers in the analyzed genomic regions, variant calling was validated by means of a separate pipeline based on widely used bioinformatics tools implemented in the Galaxy platform [31, 32]. The adopted workflow included the following steps, each one performed with ad hoc configuration: (i) sequence reads QC; (ii) removal of low-quality portions of reads; (iii) alignment to the hg19 human reference genome; (iv) selection of target aligned regions; and (v) variant calling. In details, QC was performed using the FastQC toolset [33] and reads were trimmed at the position where the evaluated median quality score dropped below 26. Alignment was performed using BWA with default parameters [34], and the resulting SAM file was converted into BAM format and sliced by selecting only reads mapping on the regions of interest specified by a BED file. Variant calling was then obtained by means of the mpileup tool implemented in the SAM tool package [35] and using default parameters coupled with a coefficient for modeling homopolymer-related sequencing errors of 40. The obtained VCF file was finally filtered maintaining only variants with quality values equal or above 100.

### Reference dataset

To perform population genetics analyses, a reference dataset made up of 836 individuals belonging to nine human groups was constructed by collecting sequence data generated by the 1000 Genomes Project [36]. In particular, populations characterized by limited genetic admixture were selected to be sufficiently representative of African (i.e., Yoruba from Nigeria, YRI; Luhya from Kenya, LKW), European (i.e., Tuscans from Italy, TSI; Utah residents with Northern and Western European ancestry, CEU; British from England and Scotland, GBR; Finnish, FIN), and East Asian (i.e., Han Chinese from Beijing, CHB; Han Chinese from Southern China, CHS; Japanese from Tokyo, JPT) variation at the examined genes.

### Population structure and differentiation analyses

Worldwide patterns of population structure at the sequenced genomic regions were explored using a pruned subset of SNPs in approximate linkage equilibrium with each other to prevent achievement of biased results for the applied multivariate analyses due to linkage disequilibrium (LD). For this purpose, the reference dataset was filtered using the PLINK package v.1.07 [37] to prune SNPs in LD by using a sliding window approach. Windows of 50 SNPs, with LD being calculated between each possible pair of SNPs, were used, and one of a pair of SNPs was removed if pairwise genotypic correlation (*r*^2^) was greater than 0.1. Each window subsequently shifted 10 SNPs forward, and the same procedure was repeated.

Discriminant analysis of principal components (DAPC) [38], which is particularly well suited for depicting genetic relationships among pre-defined groups of observations, was applied to the pruned dataset using the R *adegent* package. Once calculated principal components (PCs), DAPC was repeated with different randomized groups for different amounts of retained PCs, whose optimal number was identified as that optimizing the mean α-score (i.e., the closest to 1) obtained as the difference between observed and random discriminations. Retained PCs were passed to a linear discriminant analysis that constructed discriminant functions as linear combinations of original variables in order to show the largest between-group variance and the smallest within-group one. Given the low number of examined groups, all discriminant functions were retained to perform the analysis.

NCWS samples were subsequently represented onto the obtained discriminant functions as *supplementary individuals* (i.e., observations which do not actually participate to model construction, but which can be predicted using the model fitted on the reference dataset). In fact, NCWS data were transformed using centering and scaling of the reference dataset and according to the same discriminant coefficients as for contributing healthy individuals. Evaluation of posterior membership probabilities for both healthy and NCWS subjects to belong to a given group was finally achieved and represented in an admixture-like plot.

To point out SNPs mainly driving the observed patterns of population structure, differentiation among clusters of genetically homogeneous populations grouped according to DAPC results (i.e., Africans, AFR; Europeans, EUR; East Asians, EAS) was investigated by computing pairwise Wright’s *F*_st_ index for each SNP of the complete reference dataset. Distributions of *F*_st_ values obtained by comparing the three examined continental groups for each variant of the genome reported in the 1000 Genomes Project dataset [39] were finally used to identify SNPs at the sequenced genes showing unusual high differentiation with respect to genomic patterns (i.e., with *F*_st_ scoring in the 99th percentiles of the obtained genome-wide distributions).

### Basic descriptive and neutrality statistics

Full-gene sequence data for candidate genes pointed out by differentiation analyses were used to compute basic descriptive and neutrality statistics for NCWS and reference continental groups of populations. Accordingly, estimates of nucleotide diversity as the average number of pairwise differences (*π*), number of segregating sites (*S*), Tajima’s *D* (*D*), and Fu and Li’s *D* and *F* (*D*′, *F*) were calculated by using the DnaSP package v.5.10 [40]. Significance of these statistics was assessed by one-tailed tests aimed at comparing them with distributions of values obtained by performing 10,000 coalescent simulations conditioned on local recombination and mutation rates and assuming a neutral model of evolution. Adjusted *p* values for the adaptive Benjamini and Hochberg (ABH) procedure [41] were computed using the R package *multtest* to control the false discovery rate (FDR) at *α* = 0.01.

### Linkage disequilibrium and haplotype analyses

The PLINK package v.1.07 [37] was used to calculate pairwise LD for each SNP pair retrieved from the 1000 Genomes Project dataset [36] and included in the genomic interval covering the best candidate genes pointed out by neutrality tests, as well as their 100-kb upstream and downstream regions.

SNPs in high LD (*r*^2^ > 0.95) with the most promising candidate variants were used to statistically infer haplotypes from unphased genotypes using the Bayesian algorithm implemented in the PHASE software v.2.1 [42].

A median-joining network was used to explore evolutionary relationships among inferred haplotypes [43] by means of the Network package v.4.6.1.2 (http://www.fluxus-engineering.com). The same software was used to estimate time to the most recent common ancestor (TMRCA) using a phylogeny-based approach and a mutation rate based on the number of fixed differences between human samples and the chimpanzee, assuming 6 My as the divergence time between the two species [44].

## Results

### Sequence variability of the NCWS sample

Two amplicon-based massive parallel sequencing runs were performed on an Ion PGM™ platform to characterize profiles of 18 NCWS subjects at a 14.8-kb genomic interval including *CXCR3*, *CXCL9*, *CXCL10*, and *CXCL11* genes, as well as their promoter and untranslated regions. Respectively, 95 and 98 % of sequence reads that passed QC filters mapped to the target genes, leading to an average sequencing coverage of 407×.

A total of 48 different sequence variants were identified in the whole sample, 94 % of which were SNPs already annotated on the dbSNP database (build 137), while only 3 were small INDELs. Details of the sequence profiles for each NCWS subject are reported in Additional file 2: Table S1. On the whole, a single intronic SNP (rs2280964) was observed in seven disease individuals on the gene encoding the CXCR3 chemokine receptor that binds gliadin and represents the keystone of the investigated inflammatory pathway. This is in line with the narrow variability reported for this locus in the surveyed healthy populations pointing to remarkable conservation of its nucleotide sequence, and plausibly functional properties, also in the disease phenotype, indicating unlikely involvement in NCWS etiology (Additional file 3). Six, 18, and 23 variants were instead observed on *CXCL9*, *CXCL10*, and *CXCL11*, respectively. Both recurrent variants shared among most samples and private ones were identified (Additional file 2: Table S1), with 34 sequence changes (i.e., 32 SNPs and 2 INDELs) being present in 12 disease subjects and 14 (i.e., 13 SNPs and one INDEL) being differently shared by a minimum of 3 to a maximum of 9 individuals. Minor alleles of rs143824398 and rs35795399 were finally observed only once in the total sample.

### Population structure

To depict worldwide patterns of variation at the most informative genes (i.e., *CXCL9*, *CXCL10, CXCL11*), DAPC was applied on sequence data retrieved for nine human groups belonging to the 1000 Genomes Project dataset. When single populations were used as pre-defined groups of individuals, no clear distinction among the four EUR samples, as well as among the three EAS ones, was detectable along the first two linear discriminants that mainly described the largest between-group variance and the smallest within-group one. Only AFR populations seemed to be not completely overlapped with each other (Additional file 4: Figure S2).

A more consistent structure appeared to be evident at a lower geographical resolution, so that DAPC was repeated using continental clusters of populations as pre-defined groups of observations. Although partial overlap between clusters of samples with different origins was still detectable, as confirmed by computed posterior membership probabilities (Additional file 5: Figure S3), nearly homogeneous intra-continental genetic landscapes were observed, especially as regards EUR and EAS, together with appreciable divergence between individuals with predominant AFR, EUR, and EAS ancestries. In fact, 79 % of examined African subjects showed posterior probabilities consistent with their affiliation to AFR, while 18 and 2 % of them are clustered within EUR and EAS groups. Similarly, DAPC assigned 76 % of European individuals to EUR, whereas 17 and 7 % of them more plausibly belonged to EAS and AFR clusters. Finally, 83 % of East Asian subjects showed posterior probabilities consistent with their affiliation to EAS, while the remaining 17 % was assigned to EUR.

To contextualize NCWS genetic profiles into observed worldwide variation patterns, position of disease individuals along the computed linear discriminants was predicted using the model fitted on the reference dataset. Accordingly, 72 % of NCWS samples showed posterior probabilities consistent with their affiliation to EUR, while 28 % more plausibly belonged to the EAS cluster, a greater, but not significantly increased value in comparison with that obtained for EUR (Fisher’s exact test *p* = 0.212).

### Unusually differentiated loci

To point out SNPs that mainly drive divergence between observed clusters of genetically homogeneous populations, pairwise *F*_st_ was calculated for each nucleotide position at the examined genes and compared with genome-wide *F*_st_ distributions obtained for the same continental comparisons. Three *CXCL9* SNPs, as well as 11 *CXCL10* and 16 *CXCL11* ones, were highlighted as loci showing exceptionally high differentiation and quite similar patterns of derived allele frequency (DAF). The most outlier *F*_st_ values and the highest DAF differences (0.44–0.46) were found between EUR and EAS (Additional file 6: Table S2). Moreover, derived alleles of these SNPs turned out to be nearly absent (e.g., DAF = 0.06) or fixed (e.g., DAF = 0.94) in EAS, while at intermediate frequency (i.e., DAF ranging from 0.48 to 0.52) in both EUR and NCWS, suggesting that profoundly different demographic or selective forces have acted on these groups.

### Signatures of natural selection at the examined genes

To test whether observed DAF patterns have been actually shaped by natural selection, estimates of genetic diversity and site frequency spectrum-based statistics were computed on candidate genes pointed out by differentiation analysis using whole-gene sequence data.

After correction for multiple testing, no significant results were obtained for *CXCL9*, which showed few variants, in accordance with modest levels of polymorphism characterizing human groups of different ancestry (Table 1), and worldwide nucleotide diversity substantially lower with respect to what were observed for the other two CXCR3 ligands. Progressive reduction of variability and number of segregating sites from African to non-African populations, coupled with non-significant results obtained by neutrality tests, suggests that *CXCL9* recent evolutionary history was mainly shaped by demographic and admixture events, rather than by natural selection so that the hypothesis of the past *CXCL9*-driven adaptive events responsible for present-day NCWS epidemiological patterns could be ruled out. One-tailed tests for comparison of computed values with distributions of simulated ones instead pointed out unusual diversity and neutrality statistics for *CXCL10* and *CXCL11*, suggesting appreciable departures of their allele frequency spectra from patterns expected under neutral evolution. An exceptional increase in EUR nucleotide diversity was observed for both genes (*π* = 15.8 × 10^−4^, ABH adjusted *p* = 0.008 and *π* = 29 × 10^−4^, ABH adjusted *p* = 0.003), and comparable results were obtained also for NCWS (*π* = 16.8 × 10^−4^, ABH adjusted *p* = 0.007 for *CXCL10* and *π* = 28.9 × 10^−4^, ABH adjusted *p* = 0.0006 for *CXCL11*). Moreover, significant and largely positive Tajima’s *D* values were found for disease and healthy EUR samples for both *CXCL10* (EUR *D* = 2.608, ABH adjusted *p* = 0.008 and NCWS *D* = 2.442, ABH adjusted *p* = 0.007) and *CXCL11* (EUR *D* = 2.822, ABH adjusted *p* = 0.002 and NCWS *D* = 3.072, ABH adjusted *p* = 0.0004). Significant and consistently positive values were observed in NCWS also at *CXCL10* for Fu and Li’s *F* statistic (*F* = 2.007, ABH adjusted *p* = 0.008) and at *CXCL11* for Fu and Li’s *D* and *F* tests (*D*′ = 1.832, ABH adjusted *p* < 0.0001; *F* = 2.716, ABH adjusted *p* = 0.0003). A single result remained significant after ABH correction at *CXCL11* also for AFR (*D*′ = 1.683, ABH adjusted *p* = 0.004).

**Table 1:** Basic descriptive and neutrality statistics for the examined candidate genes

Gene	Sample	*N*	*S*	*π* (×10^−4^)	*D*	*D′*	*F*
*CXCL9* (6.4 kb)	AFR	492	40	4.5 (0.992)	−1.403 (0.007)	−0.037 (0.568)	−0.800 (0.169)
	EAS	572	18	2.8 (0.838)	−0.766 (0.158)	−0.253 (0.493)	−0.562 (0.136)
	EUR	758	16	3.7 (0.329)	0.1504 (0.621)	−1.371 (0.154)	−0.859 (0.179)
	NCWS	36	6	3.7 (0.026)	1.734 (0.025)	0.382 (0.206)	0.950 (0.176)
*CXCL10* (4 kb)	AFR	492	32	13.3 (0.259)	0.348 (0.289)	−0.643 (0.332)	−0.202 (0.440)
	EAS	572	26	6.6 (0.741)	−0.746 (0.252)	−1.146 (0.198)	−1.185 (0.136)
	EUR	758	22	*15.8 (0.009)*	*2.608 (0.006)*	0.608 (0.104)	1.772 (0.025)
	NCWS	36	16	*16.8 (0.003)*	*2.442 (0.003)*	1.266 (0.105)	*2.007 (0.009)*
*CXCL11* (3.5 kb)	AFR	492	34	24.2 (0.040)	1.827 (0.032)	*1.683 (0.006)*	2.080 (0.010)
	EAS	572	28	13.8 (0.762)	0.492 (0.247)	1.446 (0.018)	1.277 (0.082)
	EUR	758	35	*29.0 (0.005)*	*2.822 (0.002)*	−0.127 (0.466)	1.396 (0.056)
	NCWS	36	22	*28.9 (0.0006)*	*3.072 (0.0001)*	*1.832 (0.0001)*	*2.716 (0.0002)*

In brackets, *p* values obtained by 10,000 coalescent simulations conditioned on local recombination and mutation rates

Numbers in italics are the results significant after ABH correction for multiple testing to control FDR at *α* = 0.01

*N* number of chromosomes, *S* number of segregating sites, *π* nucleotide diversity, *D* Tajima’s *D*, *D′* Fu and Li’s *D*, *F* Fu and Li’s *F*, *AFR* Africans, *EAS* East Asians, *EUR* Europeans, *NCWS* Italian individuals affected by non-celiac wheat sensitivity

### Haplotype structure

LD patterns were investigated in a genomic interval covering candidate regions highlighted by neutrality tests, as well as their 100-kb upstream and downstream sequences, to detect possible functional loci located outside *CXCL10* and *CXCL11*, but responsible for the observed signatures of natural selection. A large LD block (39 kb) was found to include all SNPs showing unusual differentiation with respect to genomic patterns. When pairwise LD values were calculated, these variants turned out to be in extremely high LD (*r*^2^ > 0.95) only with each other and with none of the remaining 2898 SNPs located within the extend examined region, suggesting the highly plausible adaptive role played by some of them in EUR.

**Fig. 1:**
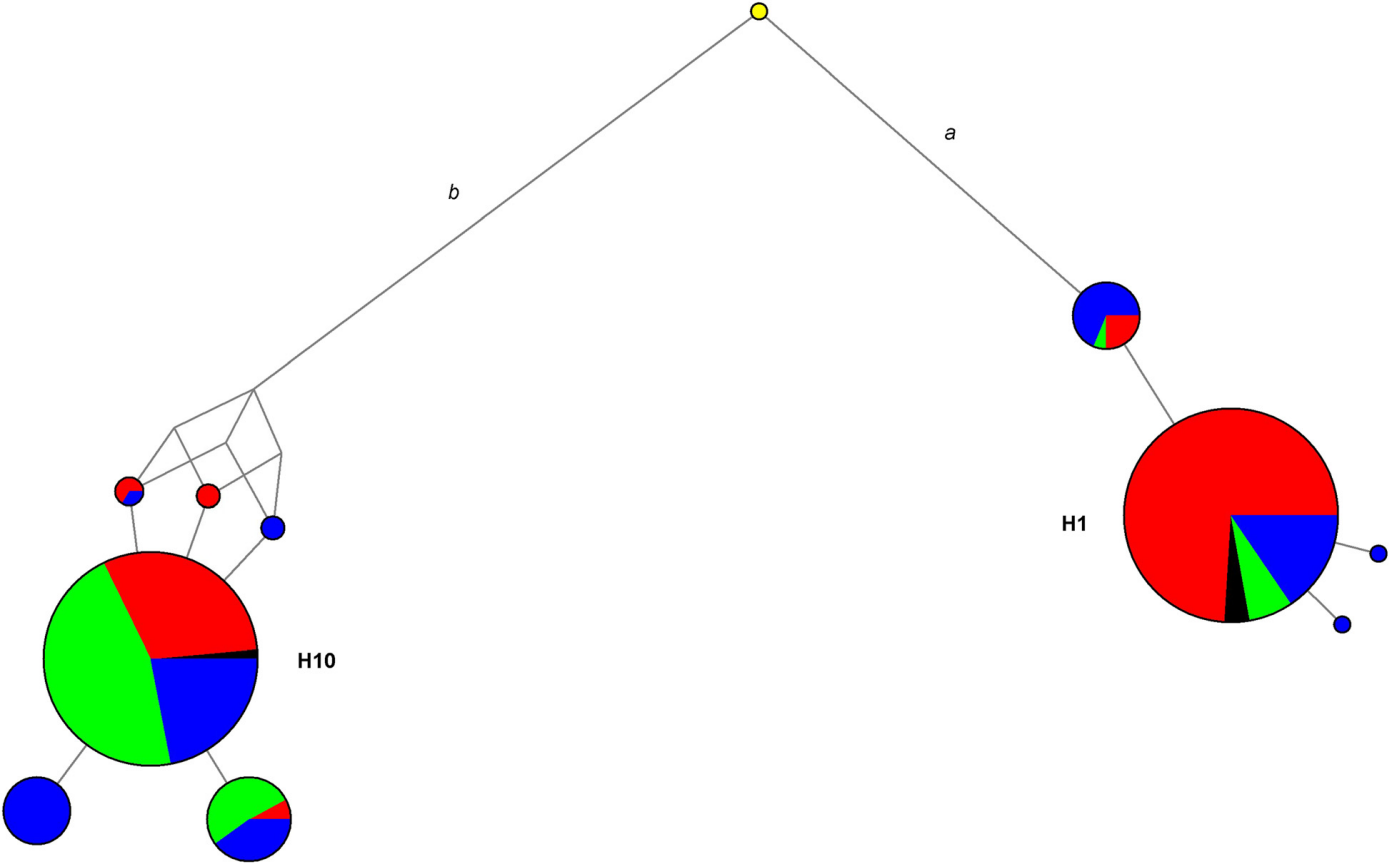
Median-joining network of haplotypes made up of SNPs unusually differentiated among continental population clusters and showing high LD (*r* ^2^ ≥ 0.95). AFR are displayed in *blue*, EAS in *green*, EUR in *red*, and NCWS in *black*. Nodes are proportional to haplotype frequencies, while branch lengths are proportional to the number of variants that occurred in the sequences

Haplotypes were reconstructed for this region of tight LD and their distributions in the studied groups were visualized by means of a median-joining network. A phylogeny characterized by ten haplotypes structured in two main clades (*a* and *b*) showing deep coalescence time (i.e., TMRCA of 1.4–1.7 My) was observed (Fig. 1). Despite overall high LD, rs4619915, rs4859588, rs4241578, and rs6819597 turned out to be recurrent, respectively, within the *a* and *b* branches of the topology, while the other three SNPs were observed in both of them. In particular, rs3921 and rs6825045 characterized all clade *a* haplotypes and the low frequency H5 and H9 clade *b* haplotypes, respectively. On the contrary, rs10025102 was found in all haplotypes of branch *b*, being present only in the singleton AFR branch *a* H3 haplotype. All these variants plausibly represented not actual homoplasies but the results of recombination or gene conversion events which occurred at the examined regions.

Most of the inferred haplotypes showed extremely low frequencies (up to 4.3 %) in all continental groups, suggesting that they have been unlikely favored by natural selection. The sole exceptions were haplotypes H1, belonging to clade *a*, and H10 belonging to clade *b*, which accounted for approximately 96 % of sampled chromosomes, being distributed in all the examined groups. In details, H1 was the predominant haplotype in EUR (50 %) and NCWS (53 %), whereas it showed moderate frequencies in AFR and EAS (21 and 6 %, respectively). It carried derived alleles at 14 SNPs, 71 % of them being located within 3′ UTR of *CXCL10* and *CXCL11* (Additional file 6: Table S2), thus potentially exerting a regulatory function on their expression. On the contrary, H10 represented the major haplotype in AFR (68 %) and EAS (92 %), reaching lower, but still remarkable frequencies in EUR (48 %) and NCWS (47 %). It carried derived alleles at the other 16 unusually differentiated SNPs, with most of them being located within intronic regions.

## Discussion

The present study investigated the genetic legacy of natural selection on genes plausibly involved in the development of NCWS to shed light on the evolutionary bases of its present-day epidemiological patterns. In fact, high disease prevalence in Western populations [3, 6] suggests that different selective pressures could have acted on its main genetic determinants in diverse human groups. In particular, it can be hypothesized that new immune-stimulatory epitopes introduced in the human diet by recent replacement of ancient wheat with modern dwarf varieties highly selected for improving industrial productivity [13, 26] turned adaptive alleles previously maintained by natural selection in the European gene pool into disadvantageous ones. This sudden dietary shift might have thus triggered a maladaptive process underlying increased NCWS susceptibility of Western people.

According to this rationale, genes pivotal in the CXCR3-inflammatory pathway were sequenced in 18 Italian NCWS subjects to carry out an explorative research not aimed at performing a traditional genetic association study, but at providing new insights into NCWS evolutionary history. We are aware of the small number of patients enrolled, nevertheless, since even limited sample sizes enable to evaluate individuals’ genetic ancestry and possible signatures of natural selection through population genetics methods, we aimed at including in this study only well-characterized disease subjects for which CD and WA were certainly excluded by immunological and clinical tests. Moreover, these patients were also selected by considering clinical onset and follow-up that clearly documented drastic resolution of symptoms after a gluten-free diet and their rapid reappearance after reintroduction of wheat in the dietary regimen. This approach enabled to detect remarkable amounts of highly polymorphic *CXCL10/CXCL11* variants in NCWS and healthy EUR, resulting in diversity levels substantially higher with respect to *CXCL9* ones (Table 1). Variation at these genes also turned out to be the main determinant of population structure depicted by DAPC (Additional file 5: Figure S3) and could be interpreted as the combined result of similar demographic processes and environmental selective pressures experienced by populations belonging to close geographical areas. Largely shared genetic background for EUR healthy and disease individuals was indeed confirmed by DAPC membership probabilities (Additional file 5: Figure S3), suggesting the absence of highly penetrant causative mutations affecting CXCR3 axis in the latter group. In particular, divergence between continental population clusters was driven by 30 high-LD common SNPs mainly located on *CXCL10* and *CXCL11* and exhibiting comparable DAFs in NCWS and EUR (Additional file 6: Table S2 and Additional file 3). Since under a neutral model of evolution demographic and evolutionary forces simultaneously affecting all loci of the genome (e.g., admixture and genetic drift) are expected to determine *F*_st_ values, these highly differentiated SNPs could be considered as variants with potential relevant functional effects and that might be subjected to differential selective pressures in diverse populations [45, 46]. In fact, null hypothesis of neutral evolution was rejected by site frequency spectrum-based tests for *CXCL10* and *CXCL11*, which showed significant excess of intermediate frequency alleles in EUR and NCWS (Table 1 and Additional file 3). Therefore, disease variation turned out to be in line with that of healthy individuals with comparable ancestry, being mainly characterized by common polymorphisms. Although paucity of rare alleles could be partly due to the limited NCWS sample size (i.e., to reduced probability to detect rare sequence changes), it might be also interpreted as a first clue suggesting that increased NCWS susceptibility is not due to deleterious variants scarcely represented in the overall population. Described departures of *CXCL10*/*CXCL11* allele frequency spectra from those expected under neutrality can result from ancient population structure or the action of balancing selection [47–50]. Despite truly significant neutrality scores were obtained almost exclusively in EUR, overall positive or moderately negative values were anyway observed also for AFR and EAS, especially for *CXCL11* (Table 1). These findings appear to be not consistent with geographically restricted population structure, rather suggesting a generalized tendency of most human groups to maintain high polymorphism most likely due to ancient selective events. More recent and stronger episodes of balancing selection could have subsequently occurred only in EUR, as previously described for other genes involved in innate immunity [51, 52], making a frequency spectrum already skewed towards intermediate alleles even more extreme and leading to the observed EUR unusual diversity and neutrality statistics. When haplotype genealogies were inferred at the region of tight LD encompassing *CXCL10* and *CXCL11* most differentiated SNPs, two highly divergent haplotype clusters were observed (Fig. 1), corroborating the hypothesis that increased EUR diversity was maintained by balancing selection [53–55]. Two common cosmopolitan haplotypes characterized these clades, accounting for the vast majority of sampled chromosomes and hence representing the most likely adaptive allelic combinations (Additional file 7: Table S3). H1 was overrepresented in NCWS (53 %) and EUR (50 %) in comparison to AFR (21 %) and EAS (6 %), whereas H10 showed the opposite pattern. While most derived alleles characterizing H10 lie within intronic regions, the great majority of those carried by H1 were located at 3′ UTRs (Additional file 6: Table S2), thus having the potential to substantially affect mRNA stability/translation efficiency and possibly leading to de-regulation of post-transcriptional expression [56, 57]. In detail, rs8878-derived allele was found to significantly enhance CXCL10 translation by increasing its mRNA half-life [18]. CXCL10 overexpression was demonstrated to be mediated also by rs3921-derived allele in patients affected by multiple sclerosis [58], invasive asperigillosis [59], and hematological malignancies [60]. Derived allele at rs3921 and *CXCL9* rs10336 also turned out to boost *CXCR3* expression, in addition to that of the respective genes, in patients with severe progression of Chagas cardiomyopathy [19]. In line with these findings, CXCL10 overexpression has been clearly proved in mononucleated cells extracted from NCWS patients exposed to wheat proteins [13]. These observations confirmed the proposed immune-modulatory role of these chemokines on the whole CXCR3 axis [61], pointing them as the master regulators of chemokine-dependent inflammatory processes. Therefore, maintenance of comparable proportions of H1 and H10 by balancing selection mainly in EUR might have been triggered by selective pressures related to the recurrent epidemics that have plagued Europeans, especially after increase in demographic density and adoption of animal domestication consequent to Neolithic transition [22]. This would have thus enabled concurrent presence in the European gene pool of both alleles able to boost inflammatory reactions and alleles responsible for moderate chemokines expression. A subtle evolutionary balance between aggressive responses to pathogens (which favor the health of individuals) and their potential side effects (which result in disease phenotypes) was thus established, enabling the maintenance of proper inflammatory defenses. Departures from this condition of equilibrium due to the disruptive impact of modern diet-related immune-stimulatory epitopes have then recently entailed increased susceptibility to inflammatory diseases for a considerable fraction of EUR subjects. Interestingly, while overall H1 frequency is slightly higher in NCWS (53 %) than in healthy Italians (TSI, 45 %), distribution of H1 homozygotes is even more different between them, with NCWS showing a more than twofold percentage in comparison to TSI. However, since the limited number of sequenced subjects prevents to perform reliable genetic association tests, genotyping experiments targeted to the most informative H1 SNPs could be carried out on a considerably larger disease sample to test an additive model for H1 contribution to increased NCWS susceptibility [62]. In fact, whether typical European H1 heterozygotes present enhanced CXCR3 axis expression with respect to H10 homozygotes, which are the most common condition in worldwide human groups, H1 homozygotes might have an even more augmented chemokine expression that could confer them a further increase in NCWS risk.

## Conclusions

The adopted anthropological evolutionary genetics perspective enabled to set NCWS variation at the CXCR3 axis into the genetic landscape of healthy individuals with comparable ancestry. Coupled with reconstruction of the evolutionary history of these candidate genes in worldwide populations, it provided an effective evolutionary medicine approach disentangling the role of different environmental/cultural factors in making populations of European ancestry more prone to develop NCWS possibly as a consequence of complex gene-environment interactions, such as that represented by recent introduction of modern wheat varieties in Western diets. Further genotyping experiments targeted to the most informative SNPs pinpointed by the present study and to additional loci involved in CD and wheat-related syndromes should be carried out on considerably larger NCWS samples to corroborate such an hypothesis, together with gene expression studies aimed at clarifying which of the examined genes actually represents the driver of CXCR3 axis overexpression in this pathological condition.

## Additional files

Additional file 1: Figure S1. Flowchart describing schematic representation of the implemented research approach detailing steps for NCWS sample selection and applied population genetics analytical workflow. (PDF 24 kb) Additional file 2: Table S1. Details of sequence profiles for each NCWS subject. (XLSX 47 kb) Additional file 3: Supplementary texts which include results, discussion, and references. (DOCX 15 kb) Additional file 4: Figure S2. First and second linear discriminants of computed DAPC specifying single populations as pre-defined groups of individuals. Populations are indicated by colors and ellipses, which model 95 % of the corresponding variability. (PDF 31 kb) Additional file 5: Figure S3. Admixture-like plot displaying membership probabilities for NCWS and continental population clusters computed by DAPC. Probabilities belonging to the EAS, EUR, and AFR clusters are, respectively, displayed in green, red, and blue. (PDF 74 kb) Additional file 6: Table S2. SNPs showing exceptionally high differentiation at the genomic level. (XLSX 11 kb) Additional file 7: Table S3. Haplogroup frequencies in the NCWS and reference samples. (XLSX 10 kb)
