# Supplementary material for: Ancient pathogen-driven adaptation triggers increased susceptibility to non-celiac wheat sensitivity in present-day European populations
Source: Genes Nutr. 2016 May 23;11:15. doi: 10.1186/s12263-016-0532-4 (PMC4968434; doi:10.1186/s12263-016-0532-4)
Supplement: Additional file 1: Figure S1. — Flowchart describing schematic representation of the implemented research approach detailing steps for NCWS sample selection and applied population genetics analytical workflow. (PDF 24 kb) [file 12263_2016_532_MOESM1_ESM.pdf]

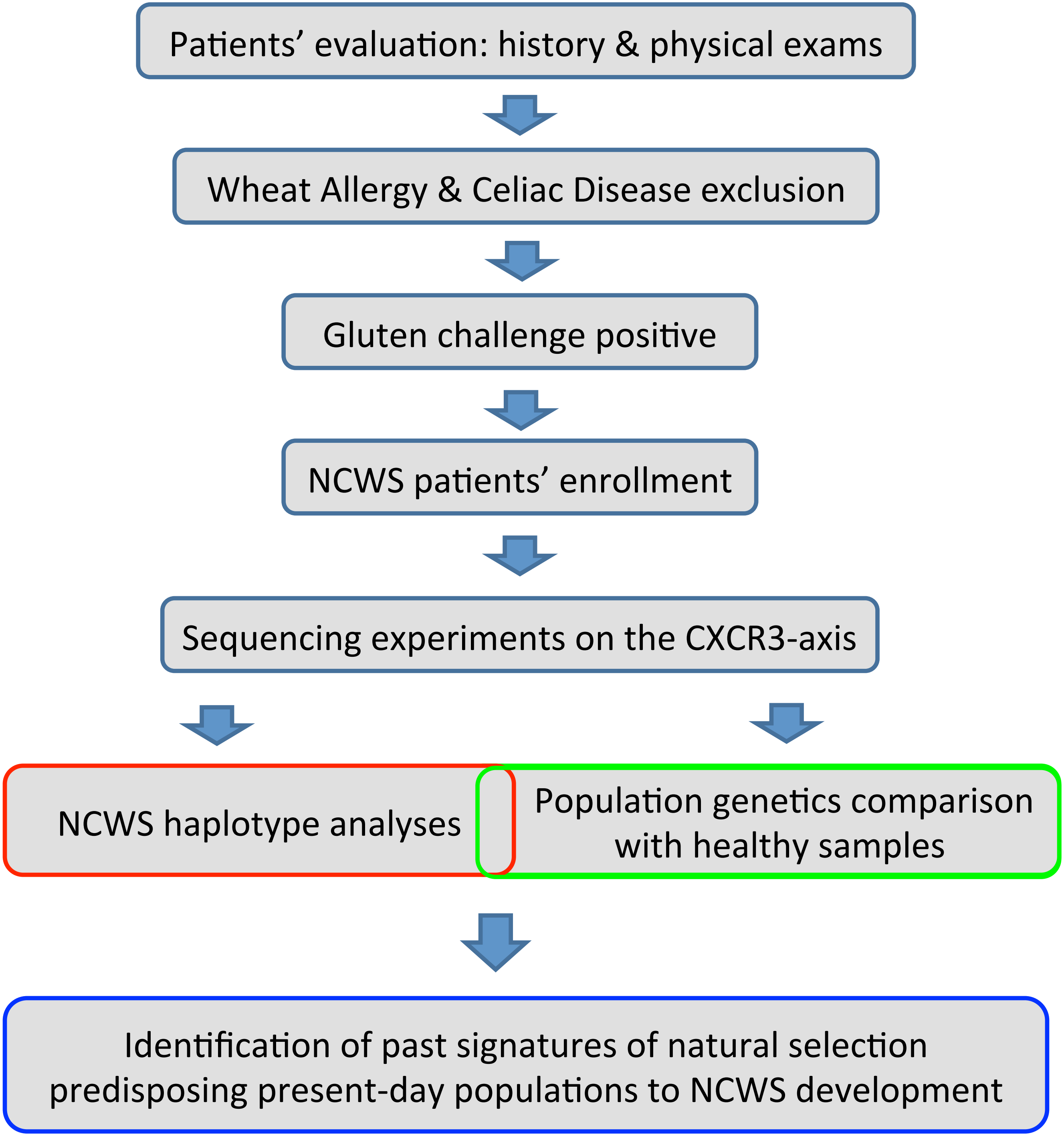
Patients' evaluation: history & physical exams
Wheat Allergy & Celiac Disease exclusion
Gluten challenge positive
NCWS patients' enrollment
Sequencing experiments on the CXCR3-axis
NCWS haplotype analyses
Population genetics comparison with healthy samples
Identification of past signatures of natural selection predisposing present-day populations to NCWS development
