# Supplementary material for: Ancient pathogen-driven adaptation triggers increased susceptibility to non-celiac wheat sensitivity in present-day European populations
Source: Genes Nutr. 2016 May 23;11:15. doi: 10.1186/s12263-016-0532-4 (PMC4968434; doi:10.1186/s12263-016-0532-4)
Supplement: Additional file 3: — Supplementary texts which include results, discussion, and references. (DOCX 15 kb) [file 12263_2016_532_MOESM3_ESM.docx]

**Supplementary Text** Includes Supplementary Results, Discussion and References.

**Supplementary Results**

The vast majority of already annotated *CXCR3* variants were fairly uncommon, with only two SNPs, in addition to the detected one, showing minor allele frequencies (MAF) considerably exceeding 5% in most human groups (Li et al. 2008; Pickrell et al. 2009; 1000 Genomes Project Consortium et al. 2010)

**Supplementary Discussion**

The observed divergence between continental clusters of populations was driven by a set of 30 common SNPs mainly located on the *CXCL10* and *CXCL11* genes and exhibiting comparable DAFs in both the NCWS and EUR samples (Table S2). By scoring in the 99^th^ percentiles of F_st_ genome-wide distributions obtained by comparing continental population groups, these loci showed extraordinarily increased levels of differentiation with respect to average genomic patterns, especially between EUR and EAS. As suggested by their quite similar DAFs, these candidate targets of selection were found to lie within a large LD block that includes also three SNPs located on the *CXCL9* gene. However, according to the above-described evidence supporting neutral evolution of this latter locus (Table 1), increased differentiation of its variants between EUR and EAS could be interpreted as a LD-related consequence of the action of natural selection on *CXCL10* and/or *CXCL11*adaptive SNPs.

Interestingly, although the performed experiments were designed to obtain high sequencing coverage in order to enable the detection of also rare nucleotide changes, NCWS variation turned out to be perfectly in line with that of healthy individuals with comparable ancestry, being mainly characterized by common polymorphisms. The capability of Fu and Li's D and F tests, as opposite to Tajima’s D, to take into account whether the observed nucleotide changes occurred in external or internal branches of the investigated genealogy (Fu and Li 1993) indeed allowed to highlight the significance of D’ and F estimates for NCWS, but not for healthy EUR, supporting a more substantial scarcity of singleton (i.e. rare) derived alleles in the former sample. The great majority of SNPs observed in the disease sample was thus recurrent among most of the sequenced subjects and at moderate to high frequency also in EUR, with the sole exceptions of two *CXCL9* and *CXCL10* singletons that showed nearly fixed ancestral alleles also in healthy populations.

While most of derived alleles characterizing H10 lie within *CXCL10* and *CXCL11* intronic regions (Table S2), the great majority of those carried by H1 were located at their 3’ UTRs, thus having the potential to substantially affect their mRNA stability and translation efficiency and possibly leading to de-regulation of their post-transcriptional expression. In particular, our results contribute to clarify that tight correlation of the effects of some of this 3’ UTRs SNPs is due to their nearly complete LD and support the hypothesis that only variation at *CXCL10* and/or *CXCL11* actually represents the driver of increased expression of these genes in a consistent fraction of European individuals. Moreover, by considering the distribution of these SNPs in a larger panel of populations than that sequenced by the 1000 Genome Project, a similar pattern could be inferred for several additional European samples and also for some worldwide scattered ethnic groups, such as Algerian Mozabites, Mbuti Pygmy from Congo, Palestinians and Balochi from the Middle East, Makrani and Kalash from the Indian subcontinent, as well as Melanesians (Pickrell et al. 2009).

**References**

Li JZ, Absher DM, Tang H, Southwick AM, Casto AM, Ramachandran S, Cann HM, Barsh GS, Feldman M, Cavalli-Sforza LL, Myers RM (2008) Worldwide human relationships inferred from genome-wide patterns of variation. Science 319:1100-1104. doi: 10.1126/science.1153717.

Pickrell JK, Coop G, Novembre J, Kudaravalli S, Li JZ, Absher D, Srinivasan BS, Barsh GS, Myers RM, Feldman MW, Pritchard JK (2009) Signals of recent positive selection in a worldwide sample of human populations. Genome Res 19:826-837. doi: 10.1101/gr.087577.108.

Fu YX, Li WH (1993) Statistical tests of neutrality of mutations. Genetics 133:693-709.
