# Supplementary figures and images for: Ancient pathogen-driven adaptation triggers increased susceptibility to non-celiac wheat sensitivity in present-day European populations
Source: Genes Nutr. 2016 May 23;11:15. doi: 10.1186/s12263-016-0532-4 (PMC4968434; doi:10.1186/s12263-016-0532-4)

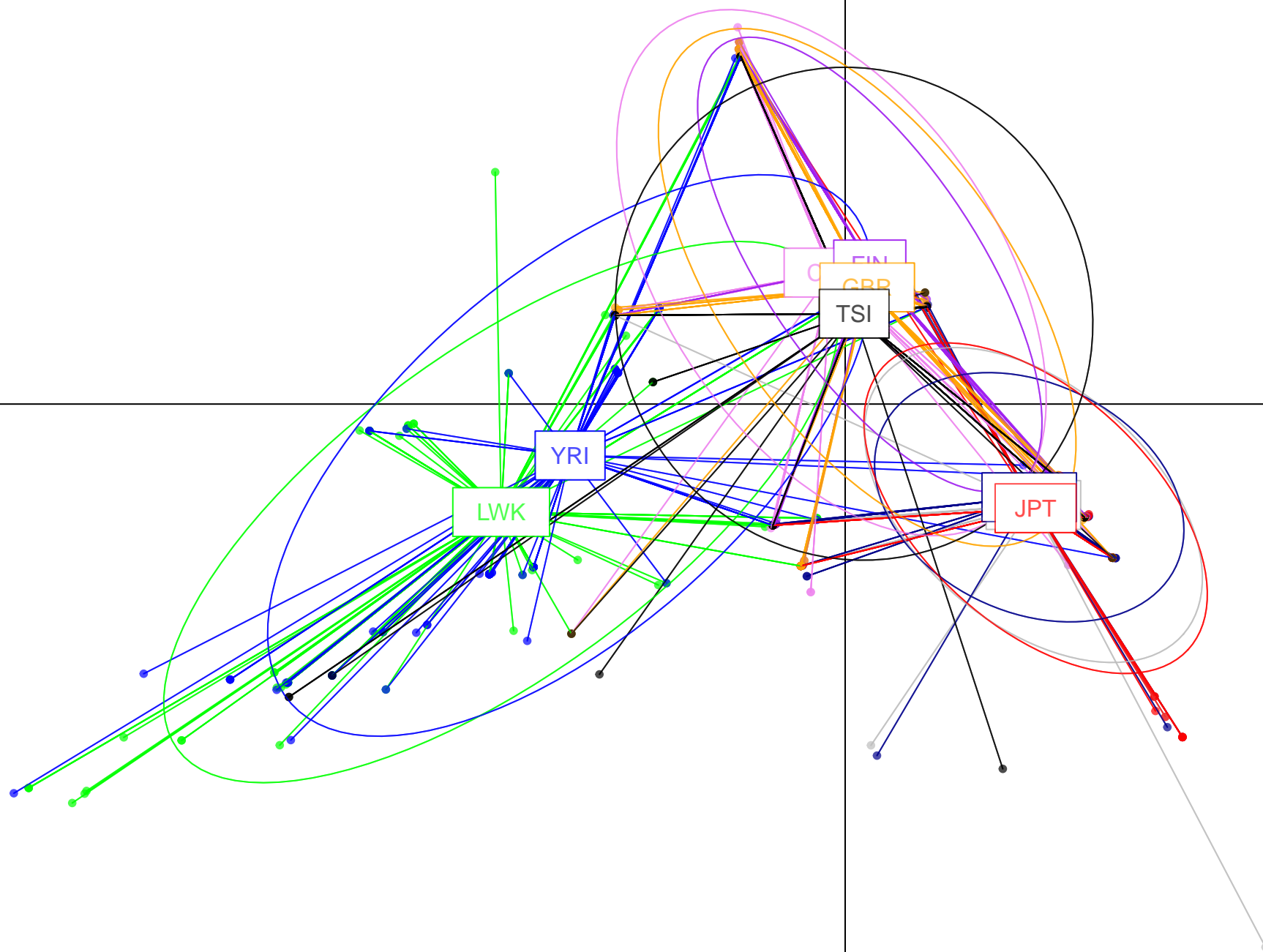

Supplement: Additional file 4: Figure S2. — First and second linear discriminants of computed DAPC specifying single populations as pre-defined groups of individuals. Populations are indicated by colors and ellipses, which model 95 % of the corresponding variability. (PDF 31 kb) [file 12263_2016_532_MOESM4_ESM.pdf]

membership probabilities

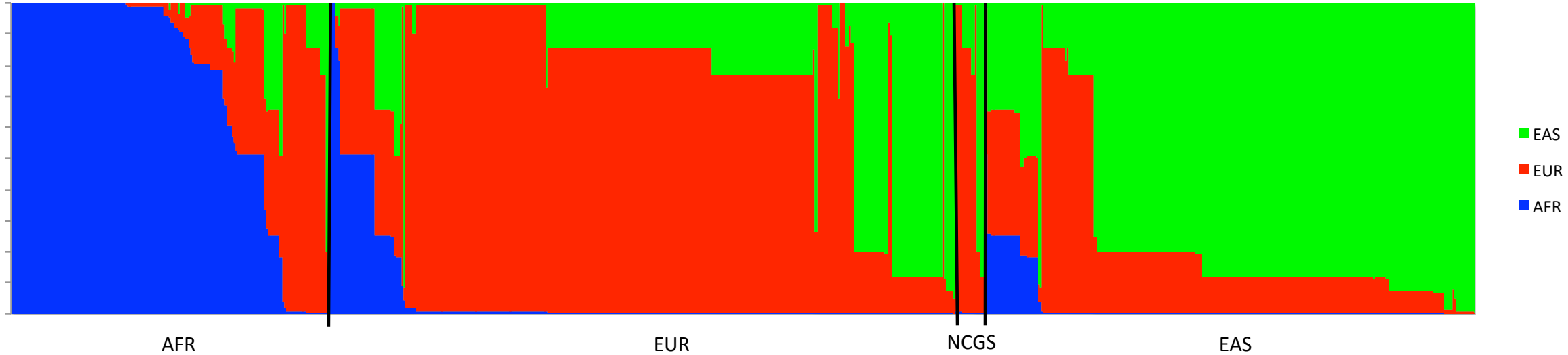

Supplement: Additional file 5: Figure S3. — Admixture-like plot displaying membership probabilities for NCWS and continental population clusters computed by DAPC. Probabilities belonging to the EAS, EUR, and AFR clusters are, respectively, displayed in green, red, and blue. (PDF 74 kb) [file 12263_2016_532_MOESM5_ESM.pdf]
